# Gut microbiota and treatment response heterogeneity across pharmacological therapies for MASH

**DOI:** 10.3389/fphar.2026.1968956

**Published:** 2026-10-20

**Authors:** Pingping Yan, Shuheng Cai, Juzhi Xia

**Affiliations:** 1 Department of Gastroenterology, Zhaotong First People’s Hospital, Zhaotong, Yunnan, China; 2 Department of Orthopedics, Zhaotong First People’s Hospital, Zhaotong, Yunnan, China

**Keywords:** gut microbiota, inter-individual variability, MASH, microbiota-derived metabolites, treatment response

## Abstract

Metabolic dysfunction-associated steatohepatitis (MASH) is a progressive liver disease with increasing global prevalence. However, substantial inter-individual variability in therapeutic response remains a major challenge for effective disease management. Although the gut microbiota is well recognized as a key contributor to MASH pathogenesis, its role in shaping pharmacological treatment response remains insufficiently understood and underexplored in clinical contexts. In this Review, we summarize evidence suggesting that gut microbiota-associated metabolites, including bile acids, short-chain fatty acids, sphingolipids, and other microbial products, may contribute to variability in therapeutic response across major pharmacological classes for MASH, including FXR agonists, PPAR agonists, FGF19/FGF21 analogues, thyroid hormone receptor-β agonists, and incretin-based therapies. However, for most therapeutic classes, direct clinical evidence linking microbiota features to inter-individual treatment response remains limited, and much of the mechanistic evidence is derived from preclinical studies. We integrate current evidence into a response-oriented conceptual framework linking microbiome composition and metabolic activity to biological processes relevant to drug efficacy. This framework identifies priorities for the prospective validation of microbiome-derived biomarkers and microbiome-informed patient stratification in MASH.

## Graphical Abstract

**Figure:**
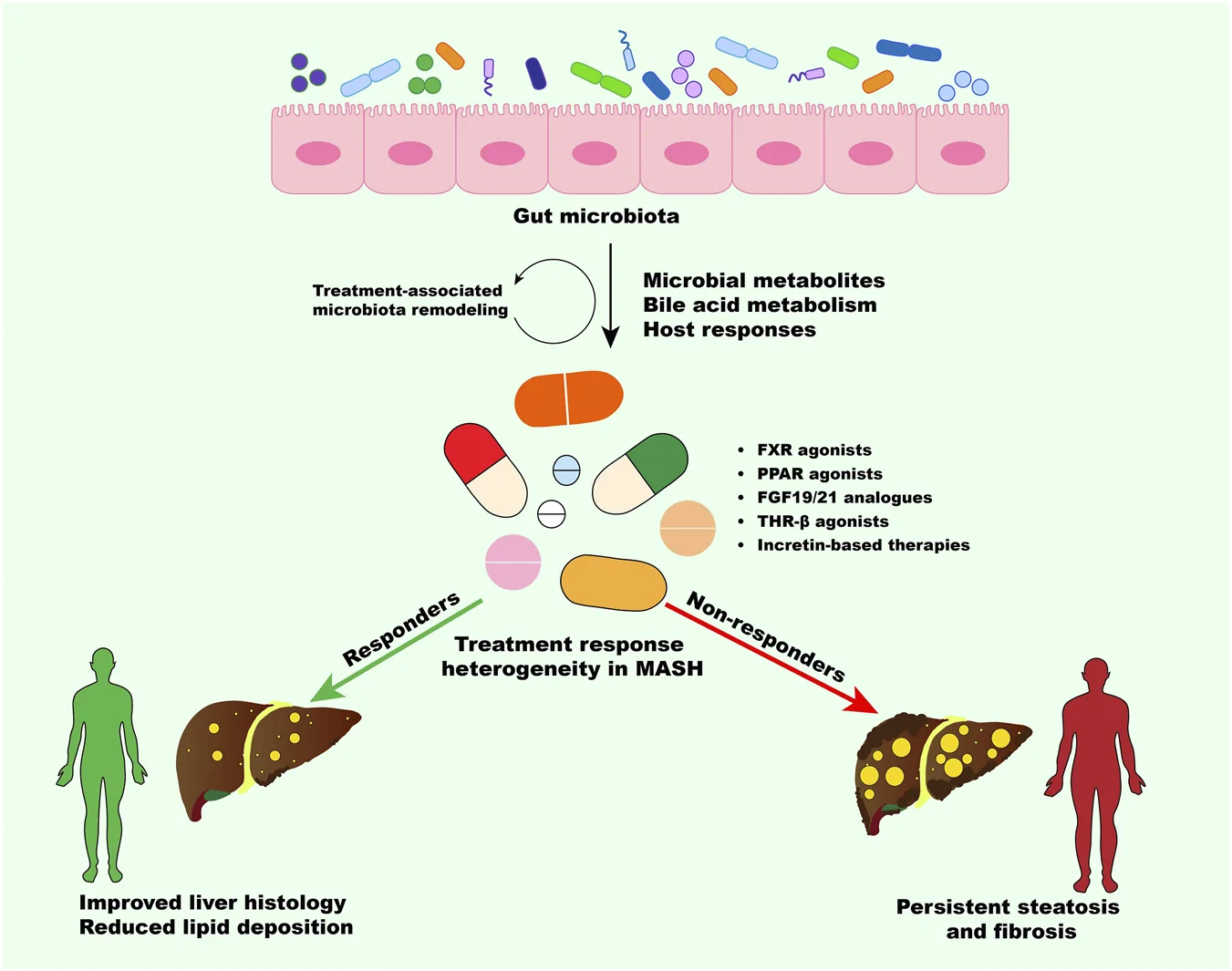

## 1 Introduction

Metabolic dysfunction-associated steatohepatitis (MASH), previously termed non-alcoholic steatohepatitis (NASH), represents the progressive inflammatory phenotype within the spectrum of metabolic dysfunction-associated steatotic liver disease (MASLD), formerly known as non-alcoholic fatty liver disease (NAFLD) (Rinella et al., 2023). MASLD has become the most prevalent chronic liver disease worldwide, affecting more than 38% of the adult population (Younossi et al., 2025). While simple steatosis is commonly reversible with lifestyle modification, a substantial proportion of individuals progress to MASH, which is characterized by hepatocellular injury, lobular inflammation, and fibrosis. Among these features, fibrosis is the strongest predictor of liver-related outcomes and the primary endpoint in therapeutic development for MASH.

The progression from MASLD to MASH is driven by multiple interrelated factors, including metabolic dysfunction, obesity, type 2 diabetes, and gut microbiota dysbiosis. Among these factors, gut microbiota has attracted increasing attention due to its capacity to modulate hepatic inflammation and metabolic homeostasis through the gut-liver axis. Gut microbiota dysbiosis can impair intestinal barrier integrity, increasing the translocation of microbial products, such as lipopolysaccharide (LPS), into the portal circulation (Di Vincenzo et al., 2024; Schnabl et al., 2025). These alterations in gut microbiota may activate hepatic immune signaling pathways, including Toll-like receptor-related signaling in Kupffer cells, thus promoting inflammation, hepatic stellate cell activation, and fibrogenesis. In addition, microbiota-derived metabolites, including bile acids (BAs), short-chain fatty acids (SCFAs), and sphingolipids, may further influence hepatic lipid metabolism and immune responses (Tilg et al., 2021). Recent evidence further suggests that gut microbiota and microbiota-derived metabolites can influence therapeutic outcomes through effects on drug metabolism, toxicity, and host metabolic pathways (Guo et al., 2026). However, despite the close relationship between gut microbiota and MASH, most studies have primarily focused on disease pathogenesis, whereas its relevance to therapeutic response remains unclear. This gap has become more significant as MASH therapy progresses toward mechanism-based pharmacological interventions targeting farnesoid X receptor (FXR), peroxisome proliferator-activated receptor (PPAR), fibroblast growth factor (FGF19/FGF21), thyroid hormone receptor-β (THR-β), and incretin pathways. Although these therapies target distinct but interconnected pathways, patients exhibit substantial variability in treatment response, particularly in outcomes such as MASH resolution and fibrosis improvement. The underlying causes of this variability remain undetermined. Specifically, it remains unclear whether pretreatment microbiome composition, microbial metabolic capacity, or treatment-induced microbiome alterations contribute to differences in therapeutic outcomes.

In this Review, we summarize current evidence on treatment-associated alterations in the gut microbiota and microbial metabolites, and discuss candidate bacterial and metabolic signatures that warrant further evaluation as potential indicators of treatment response. We further integrate evidence across five major therapeutic classes to explore how microbiome-related mechanisms may contribute to inter-individual variability in drug efficacy. The clinical development status of representative agents and the current availability of microbiota-related evidence are summarized in Table 1. Importantly, the evidence discussed in this Review spans different levels of translational maturity. We therefore distinguish findings from animal and *in vitro* models from observations in human studies. Preclinical findings are interpreted as hypothesis-generating and are not considered evidence of clinically established microbiota-mediated treatment response. Finally, we emphasize the potential of microbiome-informed strategies to support treatment stratification and precision therapy in MASH. Rather than primarily focusing on disease-associated microbiota alterations or individual therapeutic classes, this Review examines treatment response heterogeneity across major pharmacological therapies from a microbiome perspective.

**TABLE 1:** Representative pharmacological agents for MASH and the current availability of microbiota-related evidence.

Therapeutic class	Representative agents	Clinical stage	Level of microbiota-related evidence
FXR agonist	OCA	Phase III (development discontinued)	Primarily preclinical
	Linafexor	Phase II completed	Indirect or limited
	Cilofexor	Phase II (development discontinued)	Indirect or limited
	Vonafexor	Phase II (latest MASH-related study terminated)	Indirect or limited
	EDP-305	Phase IIb (development discontinued)	Indirect or limited
PPAR agonist	Elafibranor	Phase III (development discontinued)	Indirect or limited
	Rosiglitazone	Phase II (development discontinued)	Indirect or limited
	Pemafibrate	Phase II	Primarily preclinical
	Lanifibranor	Phase III (ongoing)	Indirect or limited
	Saroglitazar	Phase IIb (completed)	Indirect or limited
FGF19/FGF21 analogue	Aldafermin	Phase IIb	Primarily preclinical
	Pegozafermin	Phase III (ongoing)	Primarily preclinical
	Efruxifermin	Phase III (ongoing)	Primarily preclinical
THR-β agonist	Resmetirom	FDA-approved	Indirect or limited
	HSK31679	Phase III (China)	Primarily preclinical
	TERN-501	Phase IIa (completed)	Indirect or limited
	VK2809	Phase IIb (completed)	Indirect or limited
	ASC41	Phase II (ongoing)	Indirect or limited
Incretin-based therapy	Semaglutide	FDA-approved	Indirect or limited
	Tirzepatide	Phase III (ongoing)	Primarily preclinical

Microbiota evidence refers to studies investigating treatment-associated gut microbiota remodeling and/or the association between gut microbiota and therapeutic response. Primarily preclinical indicates that microbiota-related evidence is derived mainly from animal or experimental studies, with no or only exploratory human observations. Indirect or limited indicates that evidence is sparse or indirect and that no studies have directly evaluated the relationship between gut microbiota and therapeutic response in MASH. Clinical development status was updated as of August 2026 in this table.

## 2 Literature search strategy

Relevant literature was identified through targeted searches of PubMed and Google Scholar. Search terms included combinations of “MASH”, “NASH”, “MASLD”, “NAFLD”, “gut microbiota”, “gut microbiome”, “microbiota-derived metabolites”, “treatment response” and “therapeutic efficacy”, together with terms related to the major therapeutic classes discussed in this Review, including “FXR agonist”, “PPAR agonist”, “FGF19”, “FGF21”, “THR-β agonist”, “GLP-1 receptor agonist”, and “incretin-based therapy”. Drug-specific searches were also performed when evaluating microbiota-related evidence for individual therapies. Relevant references cited in key articles were additionally screened.

Both clinical and preclinical studies were considered when they provided information relevant to drug efficacy, gut microbiota composition or function, microbiota-derived metabolites, or potential drug-microbiome interactions in MASH. Studies not relevant to the therapeutic classes or microbiota-related questions addressed in this Review were excluded. The representative agents summarized in Table 1 were compiled separately from the literature search, and their clinical development status was checked individually using publicly available sources, including clinical trial registries, peer-reviewed literature, company disclosures, and relevant regulatory information. FDA approval status was verified using official U.S. Food and Drug Administration sources where applicable. The table was intended to provide a representative, rather than exhaustive, overview of the clinical development landscape.

## 3 Drug-microbiome interactions as a biological basis for treatment-response heterogeneity in MASH

The gut and liver are closely interconnected through bidirectional communication, a relationship commonly referred to as the “gut–liver axis”. Although numerous studies have shown that gut microbiota dysbiosis and microbiota-derived metabolites contribute to the progression from simple steatosis to MASH and exacerbate liver injury and fibrogenesis, increasing evidence suggests that the gut microbiota and microbial products may also influence pharmacological responses (Wallace et al., 2010; Haiser et al., 2014; Alexander et al., 2017; Belančić et al., 2026).

Pharmacological treatment for MASH may be accompanied by alterations in gut microbial composition and metabolic activity. Conversely, inter-individual differences in microbiota composition and metabolic capacity may shape the metabolic and inflammatory environment in which these agents act. These potential bidirectional relationships fall within the emerging field of pharmacomicrobiomics (Javdan et al., 2020; Trepka et al., 2025). Diet, age, and health status contribute to substantial inter-individual variation in the gut microbiota. Microbiota-regulated BA metabolism intersects with BA-FXR signaling; microbial modulation of lipid and energy metabolism converges with PPAR activation; gut microbiota-derived metabolites such as SCFAs and sphingolipids influence host inflammatory and metabolic pathways relevant to hepatocyte injury and fibrosis. THR-β and incretin-based therapies have also been associated with broader metabolic changes and gut microbiota remodeling. These observations indicate biological overlap between microbial functions and drug-related host pathways, but do not establish microbiome-mediated effects on drug disposition or pharmacological action.

Inter-individual variability in gut microbiota composition and metabolic capacity provides a plausible but unproven biological context for the heterogeneous therapeutic responses observed in patients with MASH. Differences in microbial communities and metabolic functions, together with treatment-associated microbiota remodeling, may influence multiple processes relevant to drug efficacy, including BA homeostasis, intestinal barrier integrity, insulin sensitivity, immune activation, and fibrogenic signaling.

Taken together, these observations support the hypothesis that gut microbiota may be involved in pharmacological response heterogeneity in MASH (Figure 1) and provide a rationale for prospectively evaluating associations between microbial features and drug-specific treatment outcomes. However, the relationships summarized here should not be interpreted as established pharmacokinetic or pharmacodynamic interactions. A microbiome-mediated pharmacokinetic interaction would require evidence that microbial activity alters drug biotransformation or systemic exposure, whereas a pharmacodynamic interaction would require evidence that microbiome features modify target engagement or downstream host responses at a given drug exposure. Such direct evidence is currently lacking for the MASH therapies reviewed here. Treatment-associated microbiota remodeling is therefore considered a separate observational phenomenon and does not, by itself, demonstrate either mechanism.

**FIGURE 1:**
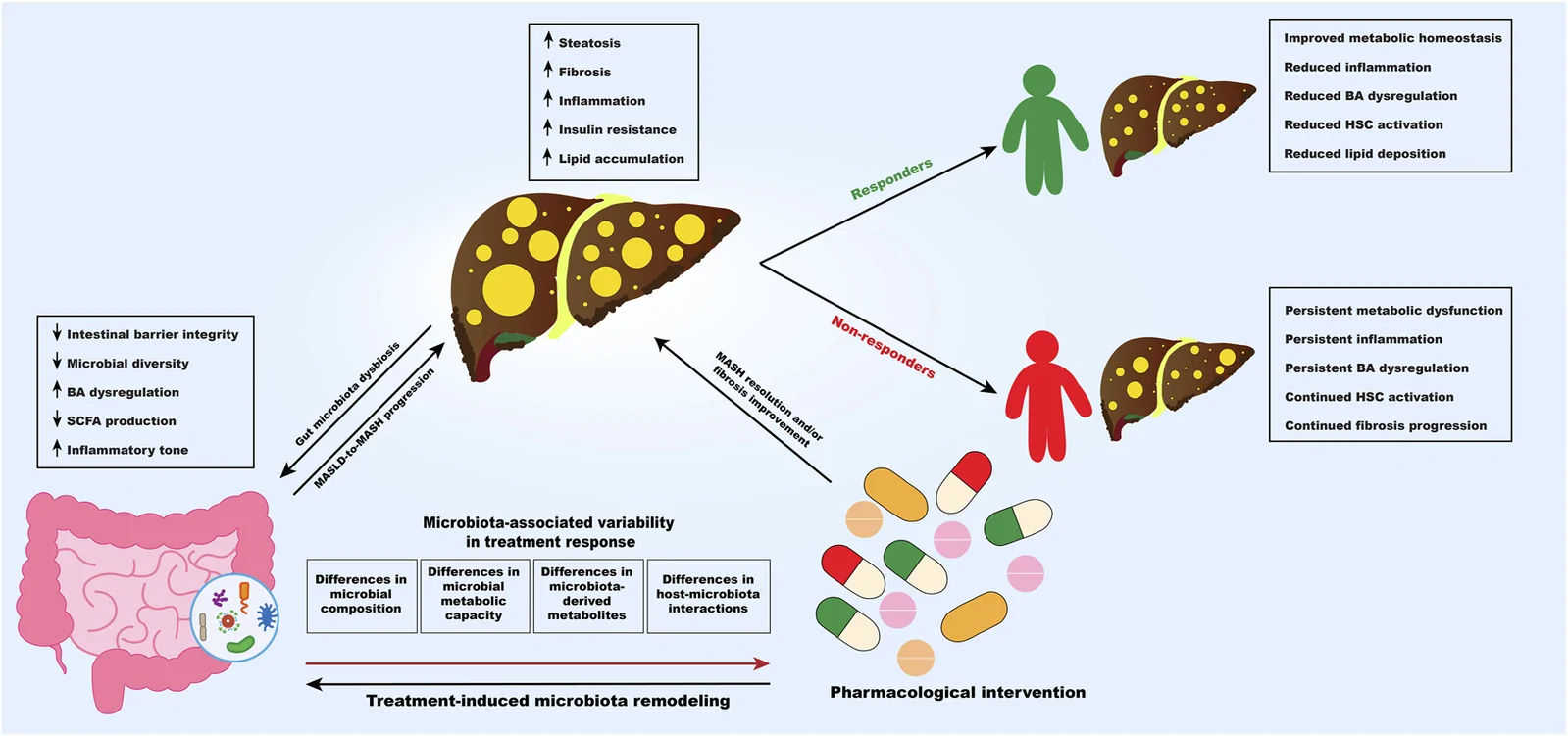
Overview of the interactions among gut microbiota, the liver, and pharmacological therapies in MASH. This figure illustrates the overall interactions among gut microbiota, the liver, and pharmacological therapies in MASH. Gut microbiota influences host metabolic and inflammatory pathways through multiple microbiota-associated mechanisms, thereby shaping the metabolic environment in which pharmacological therapies exert their effects. Pharmacological interventions may also remodel gut microbial composition and function, forming a bidirectional interaction within the gut-liver axis that may ultimately contribute to inter-individual variability in therapeutic response. (BA, bile acid; HSC, hepatic stellate cell; SCFA, short-chain fatty acid).

### 3.1 Bile acid metabolism as a mechanistic link between gut microbiota and therapeutic response

BAs are major mediators of metabolic crosstalk between gut microbiota and the host. BAs are synthesized from cholesterol in the liver through two pathways: the classical pathway, which predominantly generates cholic acid (CA), and the alternative pathway, which mainly contributes to chenodeoxycholic acid (CDCA) production. These primary BAs are conjugated mainly with glycine and, to a lesser extent, taurine in humans, then secreted into bile and released into the intestine after food intake. Within the intestinal lumen, gut microbial enzymes further transform primary BAs into secondary BAs. Most BAs are subsequently reabsorbed in the intestine and return to the liver through the portal circulation, forming the enterohepatic circulation. Accordingly, alterations in BA composition and signaling influence lipid and glucose metabolism, immune responses and fibrosis, all of which contribute to MASH progression (Chow et al., 2017; Caussy et al., 2019; Wei et al., 2024).

The composition and signaling capacity of the BA pool are jointly shaped by host biosynthesis and microbial activities. After entering the intestine, primary BAs undergo a series of microbial transformations, beginning with bile salt hydrolase (BSH)-mediated deconjugation, 7α-dehydroxylation, oxidation and epimerization reactions, generating deoxycholic acid (DCA) and lithocholic acid (LCA). These metabolic activities are primarily mediated by members of the phyla Bacteroidota, Firmicutes and Actinobacteria (Collins et al., 2023). As a result, inter-individual differences in gut microbial composition can substantially influence the abundance and diversity of circulating bile acids, thus altering BA-dependent signaling through farnesoid X receptor (FXR) and Takeda G protein-coupled receptor 5 (TGR5).

Importantly, BA metabolism may represent a common mechanistic link between gut microbiota and therapeutic response. Because microbial metabolism determines both the composition and signaling properties of the BA pool, inter-individual differences in microbial function may alter the biological context in which pharmacological agents exert their effects. Many current and emerging therapies for MASH either directly target bile acid signaling pathways, such as FXR agonists and FGF19 analogues, or act on biological processes that intersect with BA metabolism. Consequently, microbiota-dependent differences in BA composition and signaling capacity may contribute to inter-individual variability in treatment efficacy. These shared BA signaling pathways provide a biological rationale for evaluating associations between BA profiles, microbiome-derived signatures, and treatment response in MASH.

Although BA metabolism represents an important mechanism linking gut microbiota to therapeutic response, other microbiota-derived metabolites may also help to shape host metabolic and immune pathways relevant to treatment efficacy.

### 3.2 Other gut microbiota-derived metabolites shaping therapeutic response

In addition to BAs, gut microbiota produces a wide range of bioactive metabolites that influence host metabolism, immune responses, intestinal barrier function and other biological processes relevant to MASH progression and therapeutic response. Although these metabolites are generally not direct pharmacological targets, they may influence multiple biological pathways that overlap with the mechanisms of current and emerging therapies for MASH. Consequently, microbiota-derived metabolites may contribute to inter-individual variability in therapeutic response by shaping the metabolic and inflammatory environment in which these therapies act.

Among microbiota-derived metabolites, SCFAs have received considerable attention because of their broad effects on host metabolism. SCFAs, primarily acetate, propionate and butyrate, are generated through microbial fermentation of dietary fiber and regulate multiple processes implicated in MASH, including hepatic lipid metabolism, glucose homeostasis, insulin sensitivity, intestinal barrier integrity, inflammatory responses and oxidative stress (Qin et al., 2025). Several preclinical studies have reported that pharmacological interventions in rodent models are associated with increased SCFA production, accompanied by remodeling of gut microbiota, including a reduced *Firmicutes/Bacteroidetes* ratio and enrichment of *Akkermansia* (Bentanachs et al., 2024; Hao et al., 2024). However, the Firmicutes/Bacteroidetes ratio should be interpreted cautiously, as its direction and magnitude vary substantially across populations and studies are sensitive to methodological factors, including sample processing, sequencing strategy, and bioinformatic analysis. Accordingly, this phylum-level ratio is not considered a robust standalone marker of gut dysbiosis or therapeutic response (Magne et al., 2020). These observations suggest that therapeutic interventions may induce coordinated changes in microbial metabolic activities and gut microbial composition. However, these microbiota alterations are not consistent across studies. For example, obesity status has been associated with distinct gut microbial profiles in patients with MASLD, particularly in the abundance of SCFA-producing bacteria (Wang et al., 2016; Zhao et al., 2019). Together, these findings suggest that microbial SCFA production is sensitive to host metabolic status. Whether such differences contribute to inter-individual variability in pharmacological response in patients with MASH remains to be established. Compared with BAs and SCFAs, evidence linking other microbiota-derived metabolites to variability in pharmacological response remains limited. Nevertheless, available evidence suggests that metabolites such as microbial sphingolipids may influence host lipid metabolism, immune regulation and intestinal barrier function, highlighting their potential relevance to therapeutic response. Further studies are required to determine whether these metabolites could serve as candidate biomarkers or therapeutic modulators in MASH.

## 4 FXR-targeted therapies

FXR is a nuclear receptor activated by endogenous BAs and is highly expressed in hepatocytes and intestinal epithelial cells (Forman et al., 1995). Because of its central role in regulating BA synthesis, transport, and enterohepatic circulation, FXR represents one of the major molecular links between gut microbial metabolism and host metabolic regulation. CDCA is one of the most potent endogenous FXR ligands. In the liver, FXR activation induces small heterodimer partner SHP/NR0B2, which suppresses liver receptor homolog 1 (LRH-1)-mediated transcription of cytochrome P450 family seven subfamily A member 1 (CYP7A1), the rate-limiting enzyme of the classical BA biosynthesis pathway (Goodwin et al., 2000). In the intestine, FXR activation increases fibroblast growth factor 19 (FGF19) expression in humans and fibroblast growth factor (FGF15) expression in rodents. FGF19 then signals to the liver through the fibroblast growth factor receptor 4 (FGFR4)/β-Klotho receptor complex, leading to decreased CYP7A1 expression. Although both hepatic and intestinal FXR pathways ultimately suppress CYP7A1, they operate through distinct feedback mechanisms to inhibit BA synthesis. These complementary feedback pathways indicate that FXR activity is closely dependent on the composition and signaling properties of the BA pool, which is partly shaped by gut microbial metabolism. Therefore, changes in gut microbiota composition and BA-transforming capacity may influence FXR signaling and, in turn, modify the response to FXR agonists in MASH.

Obeticholic acid (OCA), a semisynthetic BA derivative and potent FXR agonist, has been extensively investigated as a therapeutic candidate for MASLD/MASH. Preclinical evidence suggests that gut microbiota composition may influence FXR-related metabolic responses. In a high-fat diet (HFD) mouse model, *A. muciniphila* and *Bifidobacterium bifidum* were shown to prevent NAFLD while modulating FXR expression and gut microbiota composition, with accompanying effects on intestinal permeability and metabolic regulation (Ottman et al., 2017; Rao et al., 2021; Wang et al., 2022). These observations support the concept that microbiota-dependent remodeling of the BA–FXR axis may alter the intestinal and hepatic signaling context in which FXR agonists act. Variations in the abundance of taxa such as *Akkermansia muciniphila* and *B. bifidum*, together with differences in microbial BA-transforming capacity, may alter the biological context in which FXR-targeted therapies act (Nian et al., 2023); however, whether these microbial features contribute to heterogeneity in clinical treatment responses remains unknown. In addition, preclinical evidence suggests that the gut microbiota may shape, and potentially influence, the therapeutic effects of OCA by modulating BA metabolism, further supporting a bidirectional interaction between FXR-targeted therapy and the gut microbiota (Liu et al., 2023).

However, the translation of these findings into clinically meaningful antifibrotic benefit remains uncertain. In a phase 3 trial, OCA was associated with fibrosis improvement without worsening of MASH, although its overall clinical utility remains limited by safety and efficacy concerns (Younossi et al., 2019).

By contrast, preclinical studies have suggested that gut microbiota-related mechanisms may attenuate the antifibrotic effects of OCA. In a western diet-induced mouse model, OCA treatment was associated with enrichment of *Bacteroides*, which increased hepatic reactive oxygen species (ROS), aggravated hepatocellular injury, and promoted hepatic stellate cell activation and fibrogenesis (Zhuge et al., 2023). These preclinical findings suggest that microbiota-related remodeling of BA metabolism, intestinal barrier integrity, and oxidative stress may modulate the antifibrotic efficacy of FXR-targeted therapies, but this possibility requires validation in human studies.

Beyond OCA, several non-steroidal and partial FXR agonists, including linafexor, cilofexor, vonafexor, and EDP-305, have also entered clinical development for MASH (Adorini and Trauner, 2023; Zang et al., 2026). However, direct evidence linking gut microbiota to therapeutic response to these FXR agonists remains limited. Overall, current evidence supports the gut microbiota-BA-FXR axis as a biologically plausible mechanism through which microbial factors could influence the biological response to FXR-targeted therapy. At present, however, this concept is supported predominantly by preclinical and mechanistic evidence, whereas direct clinical evidence linking microbiota features to inter-individual treatment response remains scarce. These findings therefore provide a rationale for evaluating microbiota-derived features as candidate biomarkers of response in future clinical studies.

Microbiome-related variation in FXR-targeted therapy is most plausibly linked to the composition and transformation capacity of the BA pool rather than to any single bacterial taxon. Clinical studies of FXR agonists should therefore test whether pretreatment serum and fecal BA profiles, together with microbial bile salt hydrolase and 7α-dehydroxylation capacity, are associated with histological response and treatment tolerability. Longitudinal sampling will be essential to distinguish pretreatment microbial features from microbiome changes that occur after FXR activation.

## 5 PPAR agonists

PPARs are ligand-activated nuclear receptors comprising three isoforms, PPARα, PPARβ/δ, and PPARγ, which regulate multiple aspects of lipid metabolism, glucose homeostasis, inflammation, and fibrogenesis (Wu et al., 2021; Sinha, 2024). In the liver, PPARα is a key regulator of fatty acid uptake and β-oxidation and also exerts anti-inflammatory effects through repression of pro-inflammatory signaling pathways (Hashimoto et al., 2000; Xu et al., 2002; Kleemann et al., 2003; Stienstra et al., 2007; Mansouri et al., 2008). The translational relevance of PPARα-targeted therapy is further supported by clinical evaluation of pemafibrate, a selective PPARα modulator, in patients with high-risk NAFLD (Nakajima et al., 2021). PPARβ/δ is expressed in hepatocytes, Kupffer cells and hepatic stellate cells, where it is involved in metabolic regulation, inflammation, and fibrogenesis (Shan et al., 2008; Liu et al., 2011; Zhang et al., 2024). The clinical relevance of PPARα/δ-targeted modulation is further supported by elafibranor (GFT505), a dual PPARα/δ agonist that has been associated with NASH resolution without worsening of fibrosis in a randomized trial (Group et al., 2016). PPARγ has attracted particular interest in MASLD/MASH therapy, and agonists such as rosiglitazone and pioglitazone have been investigated for their potential to ameliorate inflammation and fibrosis, although the role of PPARγ activation in hepatic triglyceride accumulation and steatosis remains controversial (Nan et al., 2011).

Unlike FXR, whose interaction with gut microbiota is primarily mediated through BA signaling, PPAR signaling appears to interact with gut microbiota through a broader range of microbiota-derived metabolites and metabolic pathways. Current evidence suggests that gut microbiota dysbiosis may modulate the metabolic context in which PPAR agonists act by reshaping SCFA production, intestinal barrier integrity, inflammatory tone, and lipid-derived metabolic signaling. Treatment-related microbiota remodeling has been reported following PPARα-targeted therapy. For example, in a high-fat, high-fructose rat model of steatotic liver disease, pemafibrate reversed liver steatosis and was accompanied by marked remodeling of gut microbiota and fecal BA composition, suggesting that PPAR-targeted therapy may act within a microbiota-sensitive metabolic context (Bentanachs et al., 2024). Among microbiota-derived metabolites, butyrate has been shown to ameliorate HFD-induced NAFLD through PPARα-mediated activation of β-oxidation and suppression of inflammation, supporting a functional link between SCFA signaling and PPAR-dependent metabolic regulation (Sun et al., 2018). In addition, PPAR agonists have also been associated with improvement in liver fibrosis, accompanied by remodeling of gut microbiota composition, including enrichment of *Lactobacillus* and *Akkermansia,* and alterations in microbiota-derived metabolic signaling (Wu et al., 2021; Bentanachs et al., 2024). In particular, a gut microbial metabolite of linoleic acid has been shown to mediate antifibrotic effects by inhibiting TGF-β/Smad3 signaling in hepatic stellate cells, supporting a functional link between microbiota-derived metabolites and PPAR-related therapeutic effects (Kasahara et al., 2023). SCFAs have generally been associated with protection against MASLD/MASH progression; however, reported changes in SCFA abundance following pharmacological intervention have not been entirely consistent across studies. These discrepancies may reflect differences in diet, disease stage, sampling site, and host metabolic status, which could partly contribute to differences in treatment response (Thing et al., 2024; Qin et al., 2025).

Notably, several PPAR agonists, including lanifibranor and saroglitazar, have advanced to phase II or III clinical trials and have demonstrated encouraging efficacy in patients with MASH. However, little is known about whether their therapeutic effects are accompanied by gut microbiota remodeling or whether baseline microbiota characteristics influence treatment response. Although preclinical evidence supports close and bidirectional crosstalk between PPAR signaling and gut microbiota, clinical evidence linking baseline microbiota features to responsiveness to PPAR-targeted therapy remains limited.

Collectively, available evidence suggests that PPAR-targeted therapies operate within a microbiota-sensitive metabolic environment. These findings raise the hypothesis that functional microbial outputs, including SCFA and BA metabolism, may be more relevant to therapeutic response than taxonomic changes alone; this hypothesis remains to be evaluated in clinical studies.

PPAR-microbiome interactions appear to depend on distributed metabolic functions, including SCFA production, lipid-derived metabolites, and inflammatory tone. The most informative next step would be to incorporate baseline metagenomic and metabolomic profiling into trials of lanifibranor, saroglitazar, or other PPAR agonists and test whether microbial functional pathways explain treatment response beyond conventional metabolic covariates. Such analyses may also determine whether relevant microbiome signatures are shared across PPAR agonists or depend on the specific PPAR isoforms targeted.

## 6 FGF19- and FGF21-based therapies

FGF19 and FGF21 belong to the endocrine FGF subfamily and act as key regulators of lipid and glucose metabolism. Despite sharing endocrine properties, FGF19 and FGF21 differ in their tissue origin and downstream metabolic functions. Circulating FGF19 is primarily derived from the intestine and is closely linked to BA homeostasis, whereas circulating FGF21 is mainly produced by the liver and is more strongly associated with systemic metabolic adaptation (Talukdar and Kharitonenkov, 2021). As discussed above, FGF19 suppresses CYP7A1 expression through the BA-FXR axis, which in turn reduces BA synthesis (Goodwin et al., 2000). By contrast, FGF21 primarily regulates systemic energy metabolism and insulin sensitivity, suggesting a broader interaction with host metabolism and gut microbiota.

From the perspective of gut microbiota-related treatment heterogeneity, FGF19- and FGF21-based therapies are of particular interest because they are embedded in distinct but overlapping metabolic networks that may be shaped by gut microbial composition, BA metabolism, intestinal barrier integrity, and microbiota-derived metabolites. Both FGF19 and FGF21 require β-klotho (KLB) as a co-receptor for effective activation of FGF receptor signaling; however, they differ in receptor preference and tissue distribution. In the presence of KLB, FGF19 can activate multiple FGFR isoforms, including FGFR4, whereas FGF21 shows a more restricted receptor profile, contributing to their distinct tissue-specific metabolic effects (Kurosu et al., 2007).

FGF19 and FGF19 analogues are closely linked to the BA-FXR axis. As an endocrine mediator downstream of intestinal FXR activation, FGF19 engages hepatic FGFR4/β-klotho signaling and suppresses BA synthesis via feedback inhibition of CYP7A1. In this respect, FXR agonists may be more directly governed by microbiota-derived BA ligand pools, whereas FGF19-based therapies may be somewhat less dependent on intestinal FXR activation while remaining embedded in enterohepatic signaling. Because native FGF19 has been associated with hepatocellular carcinoma risk through FGFR4 signaling, therapeutic development in MASH has mainly focused on engineered FGF19 analogues (Wu et al., 2025). Aldafermin, an engineered FGF19 analogue, has been shown to significantly reduce liver fat content, whereas evidence for fibrosis improvement remains less consistent (Harrison et al., 2021). In a prospective phase 2 study in patients with NASH, aldafermin treatment was associated with dose-dependent enrichment of *Veillonella* and changes in serum BA profiles, suggesting that *Veillonella* may serve as a marker of treatment-associated microbiome remodeling (Loomba et al., 2021). However, these findings do not establish that baseline Veillonella abundance causally determines clinical benefit.

By contrast, microbiota-related evidence for FGF21-based therapy is currently derived predominantly from preclinical models. Preclinical evidence suggests that FGF21-based therapy may act within a microbiota-sensitive metabolic environment. In a methionine-choline-deficient (MCD) diet mouse model, FGF21 treatment alleviated liver and intestinal injury, accompanied by alterations in gut microbiota composition and BA metabolism. The observation that antibiotic co-treatment produced greater benefit than FGF21 alone further suggests that gut microbiota composition may influence the therapeutic efficacy of FGF21 (Lin et al., 2023). This raises the possibility that microbial populations persisting after FGF21 treatment, or the metabolic milieu associated with them, may limit the full therapeutic response. This hypothesis remains to be validated in clinical studies. In addition, fecal microbiota transplantation from FGF21-treated mice reproduced benefits similar to those of FGF21 therapy, supporting a functional contribution of gut microbiota and BA remodeling to the therapeutic effects of FGF21 (Lin et al., 2023). These studies provide a mechanistic and preclinical basis for microbiota-related modulation of FGF21 therapy. However, whether gut microbiota contributes to differences in clinical efficacy or tolerability remains unknown.

Pegozafermin, an FGF21 analogue, is currently being evaluated in phase 3 clinical trials for MASH with encouraging efficacy signals. Treatment-emergent adverse events, particularly gastrointestinal symptoms, have been reported and may vary across dosing schedules (Loomba et al., 2023). At present, however, direct evidence linking microbiota heterogeneity to these tolerability differences remains lacking. These findings should therefore be regarded as hypothesis-generating until corresponding microbiota-response relationships are demonstrated in patients with MASH.

FGF19- and FGF21-based therapies require distinct microbiome hypotheses. In FGF19-directed therapy, the reported enrichment of *Veillonella* should be evaluated as a treatment-associated microbiome change and tested for reproducibility rather than assumed to be a baseline predictor of response. For FGF21 analogues, serial profiling of stool microbiota, BAs, and systemic metabolites in clinical trials could determine whether microbial changes precede improvements in liver fat and fibrosis or emerge secondarily to broader metabolic recovery.

## 7 THR-β-targeted therapies

Thyroid hormone signaling has been increasingly implicated in MASLD/MASH. Thyroid hormones are secreted primarily as thyroxine (T4) and are converted in peripheral tissues into the biologically active hormone triiodothyronine (T3). Thyroid hormone signaling plays a critical role in hepatic lipid metabolism, inflammation, and fibrosis (Zhou et al., 2022; Ratziu et al., 2025). Consistent with this biological role, epidemiological studies have shown that impaired thyroid function in patients with MASLD is associated with a higher risk of all-cause and cardiovascular mortality (Kim et al., 2020). These reports highlight the importance of thyroid hormone signaling in MASLD/MASH progression and provide a biological rationale for therapeutically targeting this pathway. Among thyroid hormone receptors, thyroid hormone receptor-β (THR-β) is of particular interest because it is highly expressed in the liver and has emerged as the most therapeutically relevant isoform in MASH. Recent transcriptomic and clinical analyses further suggest that THR-β-associated signaling may oppose AEBP1- and BNC2-related fibrogenic programs, and that low THR-β regulon activity is associated with hepatic decompensation risk (Kendall et al., 2023).

Resmetirom, an oral liver-directed THR-β agonist and the first FDA-approved therapy for adults with noncirrhotic MASH and moderate-to-advanced fibrosis, provides the clearest clinical anchor for this therapeutic class. In the phase 3 randomized trial, resmetirom improved key histological endpoints, including MASH resolution without worsening of fibrosis and fibrosis improvement without worsening of disease activity (Harrison et al., 2024).

Building on evidence that gut microbiota-derived sphingolipids can modulate host hepatic metabolism, preclinical analyses of HSK31679 implicated microbial glucosylceramide synthase as a mechanistic contributor to treatment efficacy (Le et al., 2022; Zhang et al., 2025). The accompanying human cohort showed treatment-associated enrichment of *Bacteroides thetaiotaomicron* and changes in microbial sphingolipid metabolism, providing exploratory translational support; however, the value of these microbial features for predicting treatment response in patients with MASH remains unvalidated. Recent commentary has therefore raised the possibility that microbiota-associated non-response may represent an important but underexplored dimension of THR-β agonist therapy (Meijnikman and Bruinstroop, 2025).

Several THR-β agonists, including TERN-501, VK2809 and ASC41, are in phase 2 clinical development for MASH and have demonstrated encouraging reductions in liver fat and improvements in liver histology. By contrast, few studies have investigated whether gut microbiota composition contributes to the efficacy of these THR-β agonists. Compared with FXR-, PPAR-, and endocrine FGF-based therapies, microbiota-related evidence for THR-β agonists remains relatively limited despite their advanced clinical development. Consequently, whether gut microbial features can be used to predict therapeutic benefit from THR-β agonists remains an important unanswered question.

Taken together, available evidence indicates that THR-β agonists have demonstrated substantial clinical efficacy in MASH, whereas microbiota-related mechanisms are only beginning to emerge. Future studies are needed to determine whether gut microbial composition and microbial metabolites influence treatment outcomes and help identify patients most likely to benefit from THR-β-targeted therapy. A key immediate question is whether the *B. thetaiotaomicron*-sphingolipid signal observed with HSK31679 is molecule-specific or reflects a class-wide mechanism of THR-β-targeted therapy. Trials of resmetirom and other THR-β agonists should evaluate baseline microbial sphingolipid biosynthetic capacity and circulating or fecal sphingolipid profiles in relation to magnetic resonance imaging-proton density fat fraction (MRI-PDFF) and histological endpoints. Replication across independent agents and patient populations would be required before these features could be considered predictive biomarkers.

## 8 Incretin-based therapies

Obesity and type 2 diabetes are closely associated with MASLD/MASH, particularly through visceral adiposity, insulin resistance and intrahepatic triglyceride accumulation, which together promote disease progression from MASLD to MASH (Saponaro et al., 2022). Accordingly, incretin-based therapies, originally developed for type 2 diabetes and obesity, have emerged as promising therapeutic strategies for MASLD/MASH. This therapeutic class includes glucagon-like peptide-1 (GLP-1) receptor agonists as well as dual- and triple-receptor agonists targeting GLP-1, glucose-dependent insulinotropic polypeptide (GIP) and glucagon signaling, thereby addressing broader metabolic dysfunction.

Incretin signaling is closely linked to BA signaling through the TGR5 pathway. TGR5 is a G protein-coupled BA receptor activated primarily by secondary BAs. Unlike FXR-mediated signaling, which primarily regulates BA synthesis and enterohepatic feedback, TGR5-mediated signaling links microbiota-derived secondary BAs to enteroendocrine L-cell activation, thus stimulating endogenous GLP-1 secretion. Activation of the BA-TGR5-GLP-1 axis contributes to the regulation of glucose and energy metabolism, insulin secretion and inflammatory responses (Wei et al., 2024). As discussed in Section 3, both microbiota-derived secondary BAs and SCFAs regulate enteroendocrine L-cell function, providing a mechanistic basis for the interaction between gut microbiota and incretin-based therapies. Conversely, pharmacological activation of incretin signaling has also been associated with changes in gut microbial composition and the intestinal metabolic environment, suggesting a bidirectional interaction between incretin-based therapies and gut microbiota (Trapanese et al., 2025; Wong and Drucker, 2025). Semaglutide, the first approved GLP-1 receptor agonist for adults with MASH and moderate-to-advanced fibrosis, has established incretin-based therapy as a major therapeutic strategy for MASH (Sanyal et al., 2025). However, studies investigating whether gut microbiota contributes to inter-individual differences in therapeutic response remain scarce.

Preclinical evidence further supports a microbiota-sensitive therapeutic context for incretin-based therapy, including changes in microbial diversity and enrichment of taxa such as *Akkermansia* and other SCFA-producing taxa. In a diabetic mouse model, tirzepatide, a dual GIP/GLP-1 receptor agonist, alleviated hepatic steatosis and was accompanied by remodeling of gut microbiota composition and BA metabolism (Hu et al., 2025). These preclinical data support the hypothesis that dual GIP/GLP-1 receptor agonists act within a microbiota-sensitive metabolic environment. Whether similar microbiota remodeling occurs in patients or contributes to inter-individual clinical response remains unknown.

Beyond monotherapy, combination strategies involving incretin-based therapies and other metabolic agents are increasingly being evaluated in MASH. For example, co-administration of efruxifermin, an Fc-FGF21 analogue, with a GLP-1 receptor agonist has demonstrated substantial reductions in liver fat together with improvements in liver injury and fibrosis, accompanied by clinically meaningful weight loss (Harrison et al., 2025). Given the physiological role of incretin hormones within the gut-liver axis, interactions between incretin-based therapies and gut microbiota are likely mediated, at least in part, through enteroendocrine signaling. However, whether combination therapies remodel gut microbiota or whether microbial characteristics influence their therapeutic benefit remains largely unexplored.

For incretin-based therapies, the central challenge is to distinguish microbiome changes related to drug action from those caused by reduced energy intake, altered gastrointestinal transit, and weight loss. Future trials should collect early and longitudinal stool samples while recording diet, body-weight trajectories, gastrointestinal symptoms, and concomitant metabolic medications, followed by mediation analyses linking microbial changes to histological outcomes. This design would help determine whether the microbiome modifies incretin treatment response or primarily reflects the systemic consequences of therapy.

## 9 Translational challenges and future directions

Most microbiome-related mechanisms reviewed here are derived from rodent models and remain hypothesis-generating because rodents and humans differ in microbial ecology, BA composition, immune responses, and drug disposition. Murine BA pools contain abundant muricholic acids, including tauro-β-muricholic acid, an intestinal FXR antagonist largely absent in humans, while mouse FGF15 is functionally related but not identical to human FGF19 (Sayin et al., 2013; Somm and Jornayvaz, 2018). These differences may influence treatment effects, particularly for FXR- and FGF19-directed therapies. Human microbiota-associated models, in which germ-free mice are colonized with microbiota from clinically characterized responders and non-responders, may help test whether response-associated phenotypes are transferable. However, incomplete microbial engraftment and the persistent influence of murine physiology limit their translational fidelity (Arrieta et al., 2016; Chiu et al., 2017). Findings from these models would therefore still require validation in longitudinal human studies.

For clinical translation, multicenter studies should harmonize biospecimen collection and storage, sequencing and metabolomic platforms, bioinformatic pipelines, and drug-specific response definitions. Association-based signatures should be distinguished from functional markers supported by convergent multiomic evidence and experimental validation. Non-responders should be treated as an informative biological subgroup. Strain-resolved metagenomic analysis, including the reconstruction and functional annotation of metagenome-assembled genomes (MAGs), could identify microbial genes and pathways associated with non-response; any resulting signals would require external replication and mechanistic validation.

Future studies should distinguish microbiome-mediated pharmacokinetic effects from pharmacodynamic effects by integrating microbial profiling with measurements of drug and metabolite exposure, target engagement, and downstream host responses. This proposed translational pathway is summarized in Figure 2. Such studies would clarify whether microbial changes alter drug disposition, modify host responses to treatment, or arise primarily as consequences of therapy.

**FIGURE 2:**
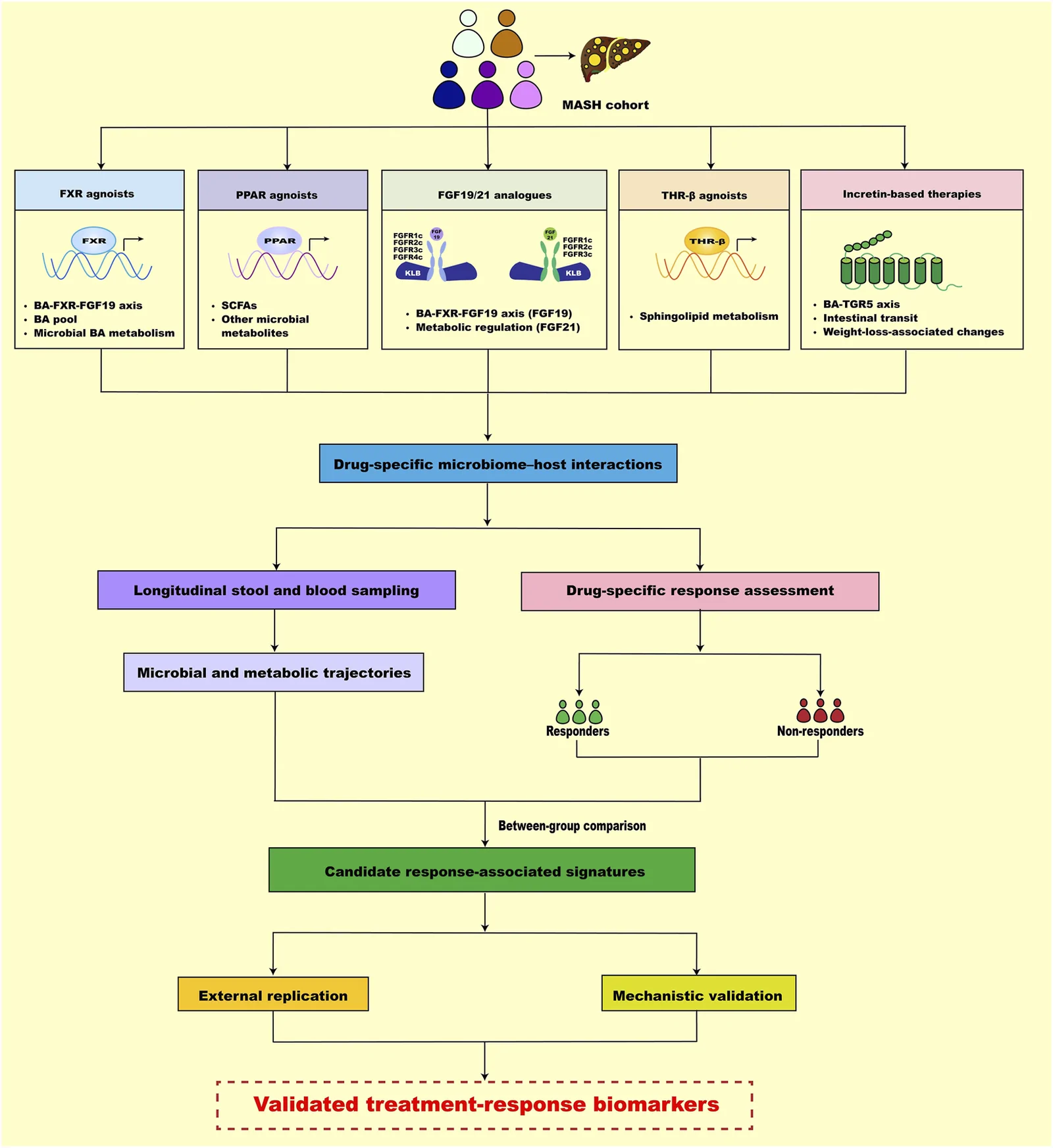
Proposed translational roadmap for the discovery and validation of microbiome-associated treatment-response biomarkers in MASH. The upper panel maps the five major therapeutic classes in MASH treatment to their principal microbiome-relevant pathways. In treatment-specific MASH cohorts, longitudinal stool and blood sampling characterizes microbial and metabolic trajectories, while drug-specific response assessment classifies participants as responders or non-responders. Integration of these data enables between-group comparisons and the identification of candidate response-associated signatures. Candidate signatures are subsequently subjected to external replication and mechanistic validation, providing a stepwise route toward validated treatment-response biomarkers. (BA, bile acid; FGF, fibroblast growth factor; SCFA, short-chain fatty acid; PPAR, peroxisome proliferator-activated receptor; TGR5, Takeda G protein-coupled receptor 5; THR-β, thyroid hormone receptor-β).

## 10 Conclusion

Overall, available evidence suggests that gut microbiota may contribute not only to MASH pathogenesis but also to inter-individual variability in pharmacological responses. Across the major therapeutic classes discussed in this Review, current evidence supports a microbiota-sensitive metabolic context in which BA metabolism, microbiota-derived metabolites, intestinal barrier integrity, and host inflammatory responses may collectively shape biological processes relevant to therapeutic outcomes. Rather than acting through a single mechanism, gut microbiota may interact with different therapeutic classes through distinct but interconnected biological pathways. However, most existing studies have focused on disease mechanisms or treatment-associated microbiota changes, whereas direct evidence linking baseline microbiota features to treatment outcomes remains limited. It remains unknown whether baseline microbiota composition or microbial metabolic signatures can predict treatment benefit across different therapeutic classes.

Given the potential bidirectional relationships between drugs and gut microbiota, treatment-associated changes in specific bacterial taxa and microbiota-derived metabolites across MASH therapies may provide clues to the biological basis of inter-individual variability in therapeutic response. In addition, gastrointestinal adverse events, including nausea and diarrhea, have been frequently reported across clinical trials of MASH therapies with distinct pharmacological targets. Whether inter-individual differences in gut microbiota contribute to variability in treatment tolerability remains unclear and warrants further investigation. Integrating microbial features with both efficacy and tolerability outcomes may therefore provide a more comprehensive basis for future treatment heterogeneity. Prospective validation in well-characterized clinical cohorts will be required before microbiome-derived signatures can support treatment stratification or individualized management in MASH.
